# Are physicians creating a barrier to pre-conception care access? A qualitative study exploring patients’ experiences and perspectives around pre-conception care

**DOI:** 10.1186/s12905-023-02820-3

**Published:** 2023-12-07

**Authors:** Lemmese AlWatban, Ebtihal Alamer

**Affiliations:** 1 Department of Family and Community Medicine, College of Medicine, King Saud University, Riyadh, Saudi Arabia; 2 University Family Medicine Center, King Saud University Medical City, King Saud University, Riyadh, Saudi Arabia; 3 Department of Family and Community Medicine, College of Medicine, Majmaah University, Al-Majmaah, 11952 Saudi Arabia

**Keywords:** Pre -conception, Pre -pregnancy, Experience, Patient -physician relationship, Access to care

## Abstract

**Background:**

The pre-conception period is an unmissable opportunity to introduce preventive measures before pregnancy to improve maternal and fetal outcomes. Despite the global pre-conception initiatives and the 2030 Saudi national vision to promote maternal, fetal health and safety, various barriers still exist. This study focuses on exploring pre-conception care extensively from the patients’ perspectives and their experience in accessing this type of care in the primary care setting.

**Methods:**

A qualitative study using interpretive thematic analysis was used to explore patients’ perspectives, and experiences in accessing pre-conception care in the city of Riyadh from January 2019 to January 2020. A semi-structured interview guide and field notes were used to collect data. A step wise interpretive and iterative process was used for data analysis and thematic extraction. Theme saturation was achieved by the eleventh interview.

**Results:**

The participants’ perspectives were influenced by their cultural beliefs, understanding of pre-conception, and their prevised barriers to approaching physicians. Three main themes emerged: A) Acceptance of pre-conception care; was heavily influenced by how they understood and defined per-conception care. B) Pre-conception health seeking behavior; demonstrated a clear disconnect between the patient and the physician. C) Expectation from health services; to raise awareness about pre-conception care and push physicians to initiate the conversation with their patients.

**Conclusions:**

An appreciable gap in the patient-physician relationship was revealed as a source of inconsistency in accessing pre-conception care. Physicians are encouraged to take the first step in demonstrating to their patients both the importance of pre-conception care and their intent to offer respectful, empathetic, and culturally appropriate care.

**Supplementary Information:**

The online version contains supplementary material available at 10.1186/s12905-023-02820-3.

## Introduction

The pre-conception period is an unmissable opportunity to introduce a set of biological, social and psychological interventions before pregnancy, to enhance the couples’ health and improve maternal and fetal outcomes [1, 2]. Despite the global pre-conception initiatives and the 2030 Saudi national vision to promote maternal, fetal health and safety, various barriers still exist [3–7]. Although barriers vary from nation to nation, misinformation about pre-conception health, social stigma and fear of the unknown are among the most frequently reported barriers in the literature [6, 8].

Previous studies have reported on patients’ expressed interest in learning about their personal pregnancy risks, regardless of being hesitant about the importance of counselling and the early introduction of folic acid in the pre-conception period [9, 10]. Moreover, other research pointed to patients feeling that their needs were under met, either because they were not offered pre-conception advice or received inconsistent advice from various healthcare professionals [11]. Patients demonstrated a preference to receiving this care from gynecologists and questioned the knowledge and capability of other healthcare professionals providing pre-conception care [10]. Consistently, several studies showed that patient acceptance of pre-conception care was influenced by the skill and expertise of the health care professional, the patient-physician relationship and the presence of family support [10–12].

To our knowledge, conclusive MOH recommendations regarding the approach to pre-conception care in Saudi Arabia (SA) are absent. Only a few local studies explored pre-conception care among diabetic patients exclusively [13, 14]. One paper we published in 2020 looked into the physicians’ viewpoints on providing pre-conception care [15]. However, this study focuses on exploring pre-conception care extensively from the patients’ perspectives and their experience in accessing this type of care in the primary care setting.

## Methods

A qualitative study using interpretive thematic analysis was chosen as it provides a deeper exploration of patients’ lived experiences and perspectives in accessing pre-conception care; especially, in developing a deeper understanding of the cultural meaning and overall essence of this shared experience [16, 17]. The principle of this design is to reduce individual experiences with a phenomenon (in this case accessing pre-conception care) to a description of the universal essence of the experience [16–18]. This description consists of “what” they experienced and “how” they experienced it, concentrating on unveiling the otherwise hidden meanings in the accounts of the experience [18].

### Participant recruitment

Recruitment was conducted through poster invitations placed throughout 10 King Saud Medical City (KSMC) primary health care centers (PHC) in the city of Riyadh, Saudi Arabia. The 10 PHC centers cover a large geographical area across Riyadh to maximize participant demographic diversity. Throughout the process of recruitment attention was given to attain maximum variation in age, gender and educational level. Participants were enrolled in the study from January 2019 to January 2020.

### Data collection

Data was collected by in-depth, semi- structured, face to face interviews. The interviews lasted approximately 30–40 min and were conducted by a researcher (EA) trained in qualitative interviews. Interviews were conducted at the most convenient primary health care center location to the participant. A semi-structured interview guide (Appendix I) and additional probes were used to explore areas in greater depth. The guide was continuously adapted to reflect adjustments from concurrent interviews and field notes.

### Data analysis

An interpretive and iterative process was used by two researchers (EA, LW) to guide data analysis. They followed a sequence of steps for data analysis and thematic extraction. Starting with, reading the transcripts and field notes independently in order to familiarize themselves with each descriptive account. Followed by identifying and labeling “units of meaning” into codes. They then met to compare, corroborate and group codes into categories within a coding template according to similarities. All categories were repetitively reviewed and revised; expanding to include new data as it emerged and collapsing to remove redundant codes. Diagrams were used to compare codes across participants Finally, categories were organized into overarching themes and sub-themes. Data analysis occurred concurrently alongside the data collection and continued until maximum variation and theme saturation (i.e. no new codes emerged) was achieved by the eleventh interview.

Since both researchers are fluent in Arabic and English and have a comprehensive understanding of the Saudi dialect and culture, coding of the data was initially done in its original language of Arabic to maintain the socio- cultural context within the text. The translation was then done for the themes and the quotes by each researcher individually. Followed by meetings to compare, agree and refine the translation. In situations where a unified translation was not reached a third colleague was consulted and invited to back-translate both versions. The resulting phrasing that was closest to the original Arabic text was then chosen [19].

### Ethical consideration and confidentiality

Prior to commencing any recruitment, ethical approval was obtained by the Institutional Review Board (IRB) of KSMC number (H-01-R-053). Involvement in this research project was completely voluntary as potential participants were only invited through poster advertising.

Written informed consent was obtained prior to all interviews and participants were assured of the privacy of the information they shared. They were reminded of their freedom to refuse to respond to any question or to end the interview at any time.

The researcher conducting the interviews was not part of the medical team caring for the participants nor were any of the physicians working at the PHC clinics involved in this research project.

All data was processed and stored through encrypted files with identifiers removed to ensure confidentiality.

### Credibility and trustworthiness of data

Multiple strategies were used to ensure the trustworthiness and credibility of the data analysis; Maximum variant sampling was used to enhance the capturing of different perspectives. All interviews were audio recorded and transcribed verbatim and were repeatedly checked for accuracy. Detailed Field notes and reflexive journaling were also completed during and immediately following each interview and were used to enhance the understanding of the text. Field notes encompassed expanded accounts of the interview, for example; the intensity of emotion expressed by the participant or any discrepancy between verbal statements and body language. The reflexive journaling accounted for the researcher’s reflections on their own thoughts and frame of mind during the interview to maximize researcher transparency and assist in identifying potential personal or professional bias. Constant referring back to the data for verification of emerging themes to ensure the applicability of the analysis to the overall context of events [20]. The researcher acting as a translator and initially coding in the original language to preserve the socio -cultural context of the analysis [19].

## Results

A total of 11 individuals participated in this study. The majority (*n* = 9) were female and two were male. Table 1 demonstrates the social and demographic features of the participants.

**Table 1:** Social and demographic features of participants

Participant (p) No.	Gender	Age	Nationality	Level of Education
p1	Female	28	Syrian	Bachelor’s degree
P2	Female	27	Saudi	Bachelor’s degree
p3	Female	28	Saudi	Bachelor’s degree
p4	Female	46	Saudi	Bachelor’s degree
p5	Female	25	Saudi	Bachelor’s degree
p6	Female	38	Saudi	Bachelor’s degree
p7	Female	45	Saudi	Bachelor’s degree
p8	Female	18	Saudi	High school diploma
p9	Female	22	Saudi	High school diploma
p10	Male	50	Saudi	High school diploma
p11	Male	26	Saudi	High school diploma

The participants’ perspectives were influenced by their awareness of pre-conception, their previous experience, and various sociocultural factors. As a result, three inter-linked themes emerged which describe the essence of patients’ pre-conception care perspectives and experience: A) Acceptance of pre-conception care, B) Pre-conception health seeking behavior, and C) Expectation from health services.

### Acceptance of pre-conception care

The degree of acceptance of pre-conception care seemed to depend on the participants’ understanding of pre-conception care. If participants defined the term pre-conception care as “a set of tests and preventive interventions that mothers and couples need to do to have a safe pregnancy and delivery” or “laboratory tests done by the mother” they were more likely to feel it was of significant value.

*“I think preconception care is what my doctor provided to me. It was a full blood test including vitamin levels. There should not be any barrier… it is a must… it should be implemented and provided…the baby’s and mother’s health are connected to each other. Every woman should care enough to show up in the clinic during and before pregnancy.” (p2).*

*“I think it is mostly advice to couples before getting pregnant…to ask for investigations to make sure that she and her husband are healthy… it is for the wellness of the mother and the baby...if the pregnancy goes well…the delivery will go well too.” (p10).*

*“No one ever mentioned preconception care to me before, but I believe it is important…for example folic acid! She should take it in advance to prevent deformities.” (p4).*

When participants felt pre-conception care was synonymous with contraceptive advice and family planning, they rejected its need, and many reported it to be a source of stress and over medicalization.

*“It is a good idea! But I feel that it is way too much…they exaggerate the need for it... like family planning…let it happen… If I plan my pregnancy and did not go as I want, I will be more anxious and unhappy about it.” (p5).*

*“I think the advantages more than the disadvantages…but it might increase concerns and fears if that person is anxious already.”(p4).*

On the other hand, noticeably, male participants felt strongly that it is purely a female issue this was evident by the fact that out of the initial 8 male patients who showed interest in the study only two took part in the interviews. The majority declined after they understood that the focus was on pre-conception care and stated “pre-conception care is only offered to women”. The two male participants that agreed to contribute to this study repeatedly expressed that their thoughts and opinions were invalid.

*“The lady, she is the one who is going to get pregnant and deal with it… but the man will suffer nothing.” fears about pregnancy are all around fears of delivery by the woman. Honestly only the woman needs more care.” (p10).*

However, even with the male participants’ evasion and the female participants’ varied definitions and lack of consensus about the value of pre-conception care, they all welcomed this type of care if offered by their physicians.

*“If the doctor offers it, it is acceptable but he should give an appropriate introduction.”(p7).*

“*I think it is better to be offered by the doctor even if i didn’t ask for it because our health status is not linear.”(p9).*

*“I have never received this care! but if the doctor offers it, I mean why not? “(p5).*

### Pre-conception health seeking behavior

**Fig. 1:**
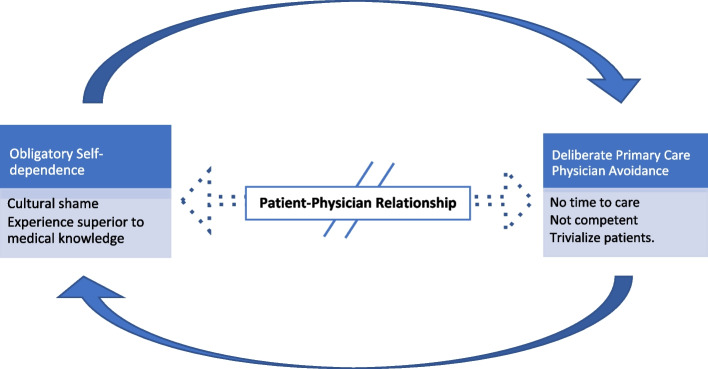
The patient-physician relationship gap resulted from obligatory self-dependence and deliberate primary care physician avoidance

The Participants’ approaches to seeking pre-conception care demonstrated a clear disconnect between the patient and physician. Two closely inter-linked sub-themes emerged that potentiate and fuel the disengagement of primary care physicians from patients’ pre-conception care: obligatory self-dependence and deliberate primary care physician avoidance (Fig. 1).

#### Obligatory self-dependence

The requisite for self-dependence in the matter of pre-conception was directly linked to the strong belief among participants in the cultural repercussions resulting from talking to strangers – including physicians – about such a private matter as conception.

*“Life is tough, so pregnancy planning is necessary, but some will not accept talking about it [pre-conception care] it is kind of a private matter when it comes to the culture.” (p1).*

*“Oh no, what shame! …It is not okay to talk about pre-conception… personally I blame both the doctors and community… these are basic principles, but we really have a low-level of awareness! … If you are not pregnant, it is shameful to talk about it…if you are not married too. Whenever we talk about it, we only hear laughs. [referring to cultural expression of shyness]” (p7).*

In addition, there was an undercurrent conviction in the superiority of personal experience over expert medical opinion and thus participants searched for information on their own or took some advice from family and friends rather than talking to a physician.

*“I search about it [pre-conception information] on my own. Sadly, this should be easy to find but it isn’t! It is a natural thing to talk about pregnancy in social gatherings and share personal experiences. Like how we talk about cooking... A common saying [in Arabic] ‘Seek advice from someone who had a similar experience, and don’t ask the doctor’ sorry I don’t like this saying, but I just wanted to clarify what I mean. Care before I get pregnant even if I am married is something I cannot talk to doctors about.” (P7).*

*“I take advice from people, from relatives who have experience… that was enough information for me so I didn’t need to see a doctor... I especially ask my sisters because we have the same genetics, for sure what happened to them would happen to me so it is best to ask them.” (p8).*

*“As I said I heard these kinds of information from relatives and close friends… and I read educational brochures about these matters.” (p6).*

Participants were also comfortable starting birth control before consulting a physician.

*“Umm…actually, I usually take a good care of my health…a relative gave me an advice regarding pills [birth control pills] and I was satisfied…I did not go to any doctor…I felt it is not worth it to go to a hospital.” (p8).*

*“I started taking my birth control pills myself… later I asked my doctor, he told me I can keep taking it.” (p5).*

Only one participant who worked in the medical field mentioned that she gained some knowledge from physicians mainly because they were available, and she could access them as friends.

*“For me…this is my general knowledge… I used to work at a hospital…some information I have got from the doctors... I also read from Wikipedia and a website belonging to the hospital I worked in.” (p4).*

The exception to prioritizing personal experience over medical recommendations was when participants reported specifically seeking medical care for infertility or to manage preexisting chronic medical conditions before pregnancy.

*“If I did not get pregnant for a long period…I will go [ to the doctor] and see what is wrong.” (p9).*

*“Last time [at the doctor], I was on cholesterol medicine and I asked if it is suitable if I got pregnant.” (p4).*

*“Umm…I’m pregnant now...before that, I went to the hospital to do some tests for my thyroid.” (p3).*

#### Deliberate primary care physician avoidance

Most participants were polite in describing their interactions with primary care physicians in the governmental Ministry of Health (MOH) PHCs. However, there was a reluctance in seeking pre-conception care through that avenue. Statements like:

*“The worst thing to me is to ask a doctor [in the primary care setting] sorry but this is what happened to me…they do not answer us properly…they do not see that I have the right to ask or know ..once a doctor answered my question by saying ‘how ignorant!’ I have the right to ask but they ignore my questions as if I have no right to ask.” (p7).*

*“The inadequacy in providing this kind of care is from the primary health care centers and the ministry of health, they have to take a step forward to promote it.” (p10).*

*“[ In primary care] so many patients are waiting, appointments are far … they cannot see and manage every patient, this is what is causing the problem.” (p2).*

*“The Governmental hospitals…not all of them will care enough… Maybe the cause is the doctor himself…if the patient did not like him or they are not convinced by his practice. Nowadays, people do not go to primary health centers because of that. There should be a doctor with a good reputation… people will talk about him and spread their positive experience. Obstetrics and gynecology mainly…I do not think general practitioners can provide pre-conception care.” (p9).*

*“I don’t think a GP or family doctor can do this. [pre-conception care] There are certain things that only obstetricians and gynecologists can deal with.” (p6).*

Suggest a lack of trust in primary care physicians working in the MOH PHCs competence in providing pre-conception care, as well as feeling trivialized and uncared for by the physicians.

Alternatively, participants who sought care from primary care physicians in the private sector were far more satisfied.

*“I do not go usually to government hospitals or health centers…but my sister goes there… she always complained about how far the appointments are…sometimes she needs to be seen before that. But it is difficult... I have health insurance so I can go to a private hospital…I consider myself lucky… but if they were able to fix this issue in government sectors… it will be a blessing for women.” (p2).*

### Expectation from health services

There was considerable agreement among participants about raising public awareness and knowledge about pre-conception care. As well as a strong push to have physicians initiate the conversation with their patients.

*“Whenever you tell any mother it [pre-conception care] is important for her baby… she will come by herself to the clinic…just raise awareness.” (p1).*

*“The doctors should be directed that if any patient comes to you, take the chance and talk about pre-conception care…If the doctor offers it. It is acceptable but he should give an appropriate introduction.” (p3).*

*“I think during premarital assessment, a doctor should counsel the couples about everything. [pre-conception, and pregnancy] There is also a limitation from the authorities…I think they should provide this care… the ministry of health and ministry of education… they should take a step to educate the young generation too.” (p10).*

Merely talking about the topic during the interview ignited a strong need among many participants to promote pre-conception care, supporting the role raising awareness plays in increasing the uptake and pursuit of such care.

*“These kinds of information, I will tell my daughters and sisters about…and anyone who is going to get married... Like please try to ask your physicians how to be ready for pregnancy without any problem…doctors are doing well…the problem lies within people.” (p4).*

*“Since you mention it, I might consider it [pre-conception care] and tell my friends and family about it.” (p9).*

## Discussion

In this study, we uncovered the role cultural beliefs play in accepting and seeking pre-conception care. Barriers from the patients’ perspectives hindering their ability to approach their physicians regarding this type of care.

An appreciable gap in the patient-physician relationship was revealed as a source of inconsistency in accessing pre-conception care. Thus emphasizing the important role that physicians play in opening the doors to patients’ acceptance and pursuit of formal pre-conception care.

Our study demonstrated the culturally driven supremacy of the woman’s own personal experience, and the advice of relatives and friends over that of a physician’s recommendations when it comes to pre-conception information. This attitude of referring to friends for pre-conception advice was also reported in Australian and UK studies [21–24].

.

To make matters more complicated, participants in our study also considered pre-conception health to be a private matter that should not be brought up with a physician, due to fear of cultural shame and breaking the social norm of concealing any pregnancy intent. Similar beliefs have been reported among Zimbabwean, South Asian, South African and Indian patients [10, 25, 26]. The literature has linked those beliefs to, the lack of husband support, difficulty accessing health care and cultural belief that showing pregnancy intent is dishonorable [10, 25, 26].

Even though the term “pre-conception care” was frequently understood as laboratory tests and preventive interventions for mothers to have a safe pregnancy and delivery, this understanding only emphasized the limited role of pre-conception care and its restriction to patients who are having difficulty conceiving or have pre- existing medical illness. Similar findings were reported as obstacles to pre-conception care in Australian, Dutch and Malaysian studies [22, 27, 28].

When participants in our study considered pre-conception care to be synonymous with family planning, they automatically rejected the need for it. Studies in Australia, the Netherlands and the US reported a similar trend and have reported that mothers-to-be believe pregnancy to be a matter of fate [22, 29, 30]. The literature also reports that pre-conception care may be perceived as over-medicalizing natural events, and thus creating a barrier to its pursuit by patients [22]. British and Irish studies have highlighted women’s fears of being lectured or judged by their physicians if they do not accept the medical advice given as a barrier to seeking pre-conception care; a similar finding to our participants [31, 32].

Many participants in this study described the primary care physicians’ inadequate approaches as part of a wider inadequacy of knowledge and training. Thus, they preferred pre-conception care to be offered by a specialist such as obstetricians and gynecologists. That is consistent with Australian and UK findings [22, 23].

The lack of trust in MOH primary care physicians and the limited visit time in the clinical setting made the participants more reluctant to approach them for pre-conception care issues. In contrast, participants expressed greater satisfaction with the private healthcare centers’ approaches to health care in general and welcomed pre-conception care in that setting. This finding has also been reported by another local study [33]. This perspective was linked to better organization, easy accessibility of care and medication availability as well as a more respectful patient-physician encounter in the private sector versus the perceived approach in the governmental sectors [33].

.Our paper points to the importance of bridging the gap in the patient-physician relationship when it comes to eliminating difficulties in accessing pre-conception care and enhancing its overall acceptance. Physicians need to take the first step in demonstrating to their patients both the importance of pre-conception care and their intent to offer respectful, empathetic, and culturally appropriate care.

Many studies have been conducted on the topic of enhancing the patient-physician relationship and have repeatedly demonstrated its value in elevating patients’ overall health and satisfaction [25, 34–37].

Our participants suggested raising awareness about the necessity of pre-conception care and encouraging physicians to pre-emptively offer it. This approach has been reported in many studies to be a successful method of promotion and improving pregnancy outcomes [21, 26, 32].

We would strongly recommend further studies exploring different strategies to strengthen the patient-physician relationship. We would urge MOH policy makers to consider better organization of clinic flow, the incorporation of health team support and pre-conception focused education to allow for better patient-centered communication.

One of the limitations of this study is that results are not universal for all health sectors in Saudi Arabia, as our sample was only from the MOH. Due to the nature of qualitative studies having smaller participant samples it would be beneficial to conduct larger quantitative studies across multiple health sectors to assess a bigger generalizability of these findings.

## Conclusion

Our research uncovered a number of culturally driven beliefs around pre-conception care and patient perceived barriers in approaching physicians for this type of care, resulting in an appreciable gap in the patient-physician relationship and contributing to the inconsistency in accessing pre-conception care. In an effort to bridge this gap and promote pre-conception care, physicians are encouraged to take the first step in demonstrating to their patients both the importance of pre-conception care and their intent to offer respectful, empathetic, and culturally appropriate care. In addition, there is a need for policy makers to promote pre-conception care and support patient-centered communication.

### Supplementary Information

**Additional file 1.**

## Data Availability

The data underlying this article cannot be shared publicly for the privacy of the participants. The data are available from corresponding authors on reasonable request.

## Abbreviations

IRB: The Institutional Review Board
KSMC: King Saud Medical City
PHC: Primary health care centers
MOH: Ministry of health
