# Supplementary material for: Are physicians creating a barrier to pre-conception care access? A qualitative study exploring patients’ experiences and perspectives around pre-conception care
Source: BMC Womens Health. 2023 Dec 7;23:651. doi: 10.1186/s12905-023-02820-3 (PMC10702085; doi:10.1186/s12905-023-02820-3)
Supplement: Supplementary file 1 — Additional file 1. [file 12905_2023_2820_MOESM1_ESM.docx]

Appendix 1

**Interview guideline**

1. Introduction and informed consent.
2. Demographic information

age, gender, nationality and level of education.

1. Tell me about your understanding of pre-conception care?

Prompts:

- - What do you think pre-conception care is?
  - What are your thoughts about this type of care?
  - How would you feel about receiving this care?

1. Tell me about your experience in accessing this type of care?

Prompts:

- - Do you feel you were offered this type of care in the past? What are your thoughts about it? How were your concerns handled?
  - How have you approached preparing for a pregnancy in the past? How did you approach family planning in the past?
  - How did you decide to seek help? Where did you seek help? What stopped you from seeking help?
  - What was your preferred information source? Why?

1. Tell me about your expectations when receiving pre-conception care?

Prompts:

- - What do you feel should be included in preconception care?
  - What do you think could be barriers to receiving this care?
  - What do you think would facilitate receiving this care?
  - Who is responsible for providing this care? What is the role of the primary care physician?
